# Exercise intensity-dependent effects of arm and leg-cycling on cognitive performance

**DOI:** 10.1371/journal.pone.0224092

**Published:** 2019-10-21

**Authors:** Mathew Hill, Steven Walsh, Christopher Talbot, Michael Price, Michael Duncan

**Affiliations:** 1 Centre for Sport, Exercise and Life Sciences, Coventry University, Coventry, United Kingdom; 2 Physical Activity & Life Sciences, University of Northampton, Northampton, United Kingdom; Teesside University/Qatar Metabolic Institute, UNITED KINGDOM

## Abstract

Physiological responses to arm and leg-cycling are different, which may influence psychological and biological mechanisms that influence post-exercise cognitive performance. The aim of this study was to determine the effects of maximal and submaximal (absolute and relative intensity matched) arm and leg-cycling on executive function. Thirteen males (age, 24.7 ± 5.0 years) initially undertook two incremental exercise tests to volitional exhaustion for arm-cycling (82 ± 18 W) and leg-cycling (243 ± 52 W) for the determination of maximal power output. Participants subsequently performed three 20-min constant load exercise trials: (1) arm-cycling at 50% of the ergometer-specific maximal power output (41 ± 9 W), (2) leg-cycling at 50% of the ergometer-specific maximal power output (122 ± 26 W), and (3) leg-cycling at the same absolute power output as the submaximal arm-cycling trial (41 ± 9 W). An executive function task was completed before, immediately after and 15-min after each exercise test. Exhaustive leg-cycling increased reaction time (p < 0.05, *d* = 1.17), while reaction time reduced following exhaustive arm-cycling (p < 0.05, *d* = -0.62). Improvements in reaction time were found after acute relative intensity arm (p < 0.05, *d* = -0.76) and leg-cycling (p < 0.05, *d* = -0.73), but not following leg-cycling at the same absolute intensity as arm-cycling (p > 0.05). Improvements in reaction time following arm-cycling were maintained for at least 15-min post exercise (p = 0.008, *d* = -0.73). Arm and leg-cycling performed at the same relative intensity elicit comparable improvements in cognitive performance. These findings suggest that individuals restricted to arm exercise possess a similar capacity to elicit an exercise-induced cognitive performance benefit.

## Introduction

There is an emerging body of multidisciplinary evidence demonstrating that regular physical activity is associated with structural (e.g. increased gray matter volume in frontal and hippocampal regions) and biological (e.g. release of neurotrophic factors and increased levels of serotonin) changes in the brain, eliciting profound benefits to cognitive functioning (e.g. attention and memory) and wellbeing in healthy young adults (e.g. better mood, reduce depression and anxiety) (see [1] for review). Consequently, a substantial body of research now exists relating to our understanding of how acute cardiovascular exercise affects cognitive performance. There is convincing meta-analytic evidence that a single bout of moderate to vigorous intensity aerobic exercise can acutely facilitate a host of cognitive functions among healthy young adults (i.e. faster reaction time) [2–5]. Several psychological and biological mechanisms have been proposed that link acute moderate intensity exercise and improved cognitive performance. From a psychological perspective, adequate levels of exercise-induced arousal may optimize the allocation of mental resources and therefore facilitate cognitive processing [3,6]. From a biological perspective, acute improvements in cognitive performance following exercise are attributed to elevated levels of brain-derived neurotrophic factor (BDNF) [7–9], increased concentrations of central catecholamines (i.e., dopamine and norepinephrine) [10,11] and increased cerebral perfusion and cerebral oxygenation [12,13]. Studies have reported either associational [9,10] or causational [14] links between changes in such physiological markers and concomitant improvements in cognitive performance among healthy young adults.

When considering potential mechanisms for any change in cognitive performance following acute exercise it is important to understand that the level of exercise induced arousal [15], the magnitude of increase in BDNF [7], concentrations of catecholamines [16] and cerebral perfusion [17], are exercise-intensity dependent. Accordingly, exercise intensity may be important as a potential mediator in the relationship between acute exercise and cognitive performance [2,5]. More specifically, for reaction time, an inverted-U effect has been reported, with moderate intensity exercise demonstrating a significantly larger mean effect size than those for low and high intensities among healthy young adults [5]. The vast majority of studies assessing the immediate effects of exercise on cognitive performance have employed stationary leg-cycling as the exercise modality. In contrast, to our knowledge, no studies have compared upper body exercise (i.e. arm-cycling) and lower body exercise (i.e. leg-cycling) effects on cognitive performance. From an exercise-intensity perspective this is surprising given that the cardiorespiratory responses to maximal and submaximal arm and leg-cycling are different [18]. For example, maximal oxygen uptake and power output are approximately 30% lower during arm-cycling than leg-cycling [18]. Consequently, submaximal exercise at the same relative intensity (i.e. 50% of maximal oxygen uptake) represents a lower absolute intensity during arm-cycling than leg-cycling. Several studies have reported that oxygen uptake, heart rate and pulmonary ventilation (i.e. physiological markers of arousal) are greater during leg-cycling compared to arm-cycling when performed at the same relative intensity [19–22]. In contrast, when performed at the same absolute power output/external workload, oxygen uptake, heart rate and pulmonary ventilation are greater during arm-cycling compared to leg-cycling [19,21,22]. Therefore, when considering mechanisms for any change in cognitive performance following exercise (e.g. increased arousal, elevated BDNF, enhanced cerebral perfusion and increased catecholamines), the mode (i.e., arms *vs*. legs) and intensity (i.e. submaximal and maximal) of exercise may be important (and interactive) for determining the amount of change in these physiological mechanisms that can be achieved. Consequently, assuming that arm and leg-cycling have the same effect on cognitive performance may lead to erroneous assumptions regarding the effect of exercise on cognitive performance. Therefore, further research is warranted in this area.

Based upon psychological and biological grounds, there is a reasonable theoretical basis for expectation that arm-cycling might elicit different effects on cognitive performance than leg-cycling. For example, during submaximal exercise with muscle groups of the upper extremity, the increase in regional cerebral blood flow is greater than for comparable exercise with the lower extremity [23,24]. Importantly, greater cerebral blood flow during exercise appears to increase neuronal activity in the prefrontal cerebral cortex, which in turn elicits an improvement in executive function [25]. There is also evidence that moderate intensity (50% maximal oxygen uptake) arm-cycling (unfamiliar exercise) leads to a greater alpha activity in the frontal brain regions, while leg-cycling (familiar exercise) leads to an increase in alpha activity in the parietal cortex [26]. This is important because executive function tasks require more in the way of prefrontal cortex activation than other tasks [27], and improvements in executive function after acute aerobic exercise are associated with increased activation in the prefrontal cortex [28]. There is also evidence that arm-cycling may elicit a greater catecholamine output compared to leg-cycling at a given oxygen uptake [29]. During maximal incremental exercise, mental effort is lower during arm-cycling compared to leg-cycling (as deduced by a reduction in the cerebral metabolic ratio) [23]. Therefore, executive functioning might be affected differently according to the active musculature (arms vs. legs) and intensity (maximal and relative/absolute submaximal) of exercise. Although these hypotheses still need to be empirically tested, there is reasonable theoretical basis for different improvements in post-exercise cognitive performance as a consequence of arm compared to leg-cycling. Examining the effects of arm exercise on cognitive performance in healthy young adults will have important implications in and may lead to the development of recommendations of exercise interventions for populations restricted to upper body exercise (e.g., lower limb orthopaedic problems, neurological disorders or peripheral arterial disease).

To date, the effects of arm and leg-cycling on cognitive performance have not yet been compared, and it is therefore not possible or appropriate to generalize any findings derived from leg-cycling into arm-cycling. Given that no study has examined whether differences exist between arm and leg-cycling, we propose to elucidate whether arm-cycling has different effects on cognitive performance to leg-cycling at maximal and submaximal intensities. Submaximal exercise protocols were matched for (1) relative (% of maximal power output) and (2) absolute (identical power output) intensities. Based on the reviewed literature, our hypotheses are as follows; (1) improvements in executive function (reaction time [ms] and accuracy [%]) will be greater following arm-cycling than leg-cycling at the same relative intensity, (2) arm-cycling will elicits greater improvements in executive function than leg-cycling at the same absolute intensity, (3) maximal arm and leg-cycling would elicit poor performance (i.e. slow reaction time and reduced accuracy) in cognitive function post-exercise.

## Materials and methods

### Participants

An a priori power analysis (statistical power = 0.80, alpha = 0.05, effect size = 1.7) was conducted for speed of processing during incongruent conditions [28], and revealed that 5 participants would be sufficient for finding statistically significant effects of acute exercise on cognitive performance. However, meta-analyses have reported that the effect size’s for acute exercise and speed of processing are heterogeneous and only small to moderate (Hedges g; 0.30) [5]. Therefore, we took a cautious approach to our power analysis and used an effect size of d = 0.9, which revealed that 12 participants would be sufficient for finding statistically significant effects of acute exercise on cognitive performance. Therefore, a convenience sample of thirteen non-specifically trained males (age, 24.7 ± 5.0 [18–31] years; mass, 74.1 ± 9.4 kg; height, 1.77 ± 0.08 m; body mass index [BMI], 23.58 ± 2.63 kg.m^-2^; cycling maximal oxygen uptake, 44.3 ± 7.4 ml/min/kg) gave written informed consent prior to participation. All participants were physically activity and accustomed to regular sports training (team sports) 2 to 3 times per week for a mean of 7.2 ± 3.7 h/week. Physical activity levels were ascertained using an in-house health screening questionnaire. Only males were included due to potential gender differences in upper body exercise capacity which may have impacted on the inter-individual differences in fitness level that might cause a high variability between individual exercise intensities and/or cognitive responses. The experimental procedures were carried out in accordance with the standards outlined in the declaration of Helsinki (1964) and the study received approval by the University of Northampton research ethics committee. Participants completed the Physical Activity Readiness Questionnaire (PAR-Q) to detect potential risk factors that might affect their ability to exercise safely. All participants reported being right-handed and did not wear corrective lenses. Inclusion criteria were age (18–35 years). Exclusion criteria were BMI > 30, self-reported history of psychiatric, neurological, cardiovascular or pulmonary diseases, orthopaedic pathology or musculoskeletal dysfunctions.

### Experimental design

**Fig 1:**
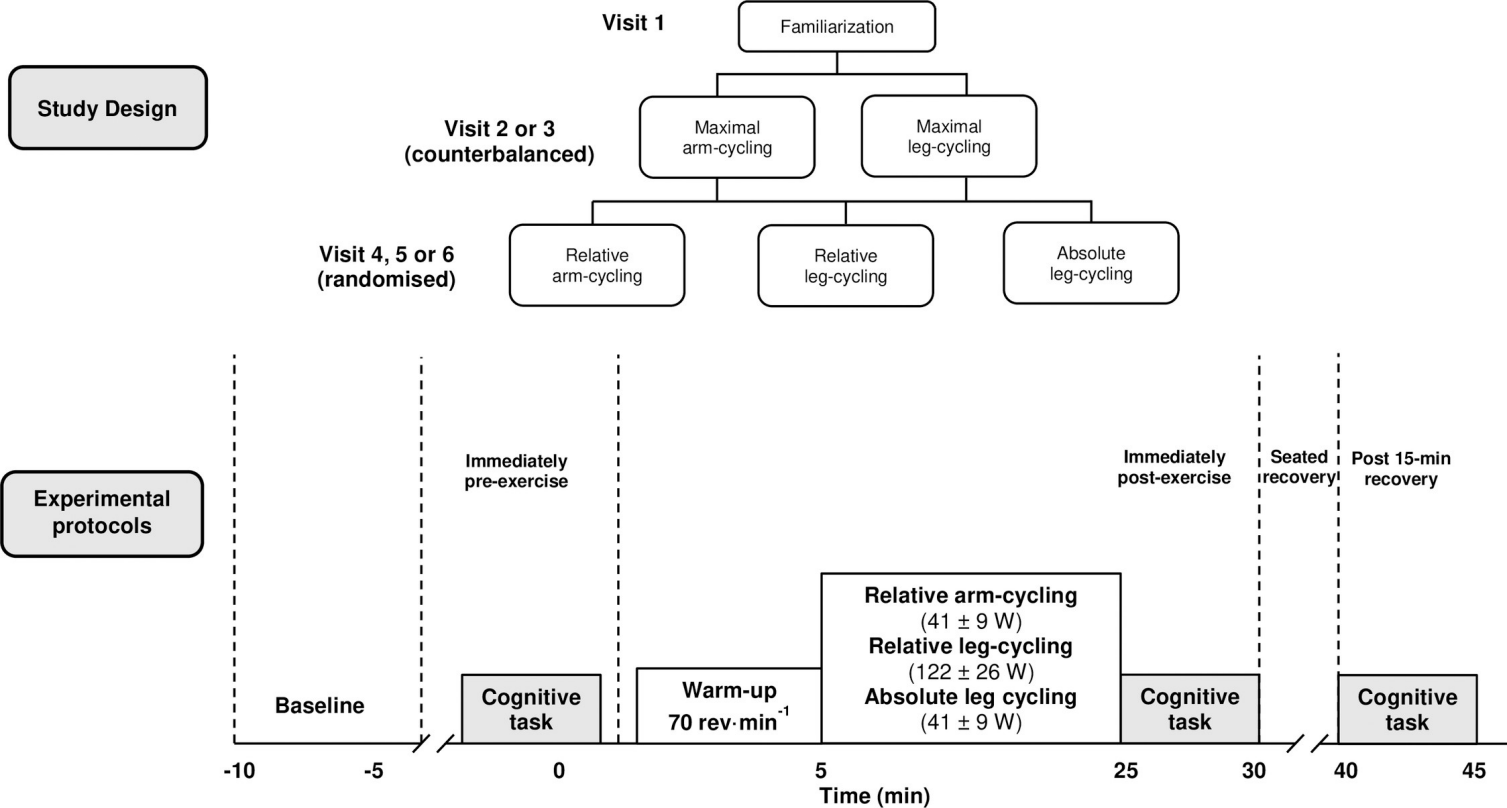
Schematic of the experimental design and experimental protocols.

This study employed a repeated-measures design. All participants attended the laboratory on six separate occasions (Fig 1). On the first occasion (visit 1), participants underwent familiarization to the cognitive test to minimize potential learning effects and attempt to achieve a consistent level of reaction time and accuracy performance. The first visit also served as a habituation test to familiarize participants to arm-cycling. On two separate occasions (visits 2 and 3), participants completed incremental exercise tests on both an arm-crank ergometer (arm-cycling) and a cycle ergometer (leg-cycling), which served to determine the maximal oxygen uptake ($\dot{V}O_{2\max}$) and exercise workloads for subsequent experimental tests. Maximal tests were also used to determine the effects of exhaustive arm and leg-cycling on cognitive performance. Exercise tests were completed in a counter-balanced order. Both tests consisted of an incremental protocol on a mechanically braked ergometer (Monark, 824E, Ergomedic, Sweden), and were completed at the same time of day to account for circadian rhythm effects, but separated by a minimum 72 h. On three final occasions (visits 4, 5 and 6), participants undertook 20 min steady-state submaximal exercise as follows: (1) relative intensity arm-cycling at 50% of the ergometer specific maximal power output (*W*_max_), (2) relative intensity leg-cycling at 50% of the ergometer specific *W*_max_, (3) absolute intensity leg-cycling at the same absolute power output as relative intensity arm-cycling. The order of exercise trials were randomized. The Erikson Flankers executive function test [30] was administered before the start of, immediately after and 15-min after each exercise intervention. Participants were asked to refrain from physical activity and caffeine/ alcohol consumption 12 hr prior to testing. Participants were also asked to not eat 3 hr prior to maximal arm and leg-cycling tests.

### Cognitive task procedure

Before, immediately after and following a 15-min recovery from exercise, all participants completed a modified version of the Eriksen Flanker test [30,31] to assess cognitive performance. The Eriksen Flanker test has been widely used test to assess the effects of exercise on cognitive performance [31–33]. Participants were instructed to respond as quickly and accurately as possible to a target presented centrally on a computer screen at eye level and at a distance of ~1m. Stimuli consisted of five arrowheads presented horizontally, which were equally likely to point to the left (i.e. < < < < <) or right (i.e. > > > > >). The flanking stimuli (i.e. the four arrowheads surrounding the central placed target stimulus) were equally likely to be congruent (50%) (i.e. same direction; < < < < <) or incongruent (50%) (i.e. opposite direction; > > < > >) with the central target arrow. When ‘<‘ was the target stimulus, participants responded with their left index finger. When ‘>‘ was the target stimulus, a right index finger response was required. Participants were asked to ignore the flanking arrowheads. A total of 30 practice trials were administered prior to the start of testing in line with prior procedures [31,32]. Stimuli consisted of white arrows on a black background, with each target measuring 2.5 cm in height and 1.8 cm in width. Each test involved five blocks of 100 trials where stimuli were presented for 100 ms with a response window of 1000 ms and an inter-stimulus interval of 1500 ms [34]. Total task duration was ~3 min. This task allowed for the calculation of reaction time (ms) (i.e. the time interval between stimulus onset and time of response button pressing) and response accuracy (%). Before the experimental sessions (visit 1), participants undertook a familiarization session consisting of 8 blocks of 64 trials. Mean and standard deviation (SD) reaction time was 420 ± 28 ms and 470 ± 33 ms for congruent and incongruent conditions, respectively. Response accuracy was 99 ± 1% and 94 ± 5% for congruent and incongruent conditions, respectively.

### Maximal graded exercise tests

The leg-cycling protocol started at a power output of 70 W with increments of 35 W every 3 min until volitional exhaustion. The arm-cycling protocol involved an initial power output of 35 W, with increments of 20 W every 3 min until volitional exhaustion [19]. For the arm-cycling trial, the ergometer was clamped onto a sturdy table and foot pedals were replaced with pronated-position hand grips. The ergometer was height-adjustable which enabled the crank axis to be aligned with the center of the glenohumeral joint. Arm-cycling trials were performed in a seated position (knees flexed to 90°) without torso restraint. A cadence of 70 rev·min^−1^ was employed throughout both trials. Expired gas was analyzed using a breath-by-breath online gas system (MetaMax, Cortex Biophsik, Borsdorf, Germany) for oxygen uptake ($\dot{V}O_{2}$) and pulmonary ventilation (${\dot{V}}_{E}$). We also calculated breathing frequency (B_f_) as a marker of physical effort [35]. Expired gas data were averaged over the final 30 sec of each incremental stage and prior to reaching volitional exhaustion. Heart rate (HR) was continually monitored (Polar Electro, Oy, Finland) and recorded in the final 10 s of each incremental stage and immediately upon reaching volitional exhaustion. A rating of perceived exertion for both local (working muscles; RPE_L_) and central (cardiorespiratory; RPE_C_) using the 6–20 point Borg scale [36] was obtained at the same time as HR and immediately upon reaching volitional exhaustion. Heart rate was also recorded during the performance of the cognitive tasks.

### Submaximal exercise tests

Two submaximal trials involved participants exercising at 50% of their ergometer specific *W*_max_ (relative intensity arm-cycling; 41 ± 9 W and relative intensity leg-cycling; 122 ± 26 W, respectively). Due to lower the *W*_max_ achieved during maximal arm-cycling, a third trial was performed on the cycle ergometer at the same absolute power output as the 50% *W*_max_ arm-cycling trial (absolute intensity leg-cycling; 41 ± 9 W). Prior to all trials, participants were required to perform a 5 min warm-up on the unloaded ergometer at a cadence of 70 rev·min^-1^. Expired gas, heart rate and ratings of perceived exertion were obtained in 5 min intervals during each exercise trial. As with the maximal tests, HR was also recorded during the performance of the cognitive tasks. All test sessions took place between 9:00 h and 11:00 h (morning session) and 13:00 h and 15:00 h (afternoon session). For each participant, maximal and submaximal exercise tests were completed at the same time of day (± 1 hour) to control for physiological variation due to circadian rhythms.

### Statistical analyses

Data were analyzed using SPSS version 25.0 (IBM Inc., Chicago, IL). For all analyses, normality (Shapiro–Wilk Test) and homogeneity of variance/sphericity (Mauchly Test) were checked prior to undertaking parametric tests. Separate two-way analysis of variance (ANOVA) with repeated measures on both factors (e.g. *trial*; arm-cycling vs. leg cycling × *time*; pre, immediately post and 15 min post exercise) were conducted to examine changes in dependent variables (reaction time and response accuracy) during congruent and incongruent trials. Maximal and submaximal tests were analyzed separately. Separate two-way analysis of variance (ANOVA) was conducted to examine differences in physiological and perceptual responses between maximal (arm *vs*. leg-cycling) and submaximal (relative arm-cycling *vs*. relative leg-cycling *vs*. absolute leg-cycling trials. To account for differences in the duration of the incremental arm and leg-cycling tests, physiological and perceptual responses were compared at the same isotime points (20, 40, 60, 80, 100% of the end ergometer-specific exercise time) (e.g. *trial*; arm-cycling vs. leg cycling × *isotime;* 20, 40, 60, 80, 100%). Post-hoc analyses with the Bonferroni-adjusted *α* for multiple comparisons were conducted to follow up significant effects. For ANOVA’s, effect sizes are reported as partial eta-squared value (*η*^2^) where appropriate. Cohen’s *d* effect sizes are reported for pairwise comparisons and were interpreted as trivial (0–0.19), small (0.20–0.49), moderate (0.50–0.79) and large (≥0.80) [37]. All values are expressed as mean ±SD. The alpha value was a priori set at p ≤ 0.05.

## Results

### Maximal physiological responses

**Fig 2:**
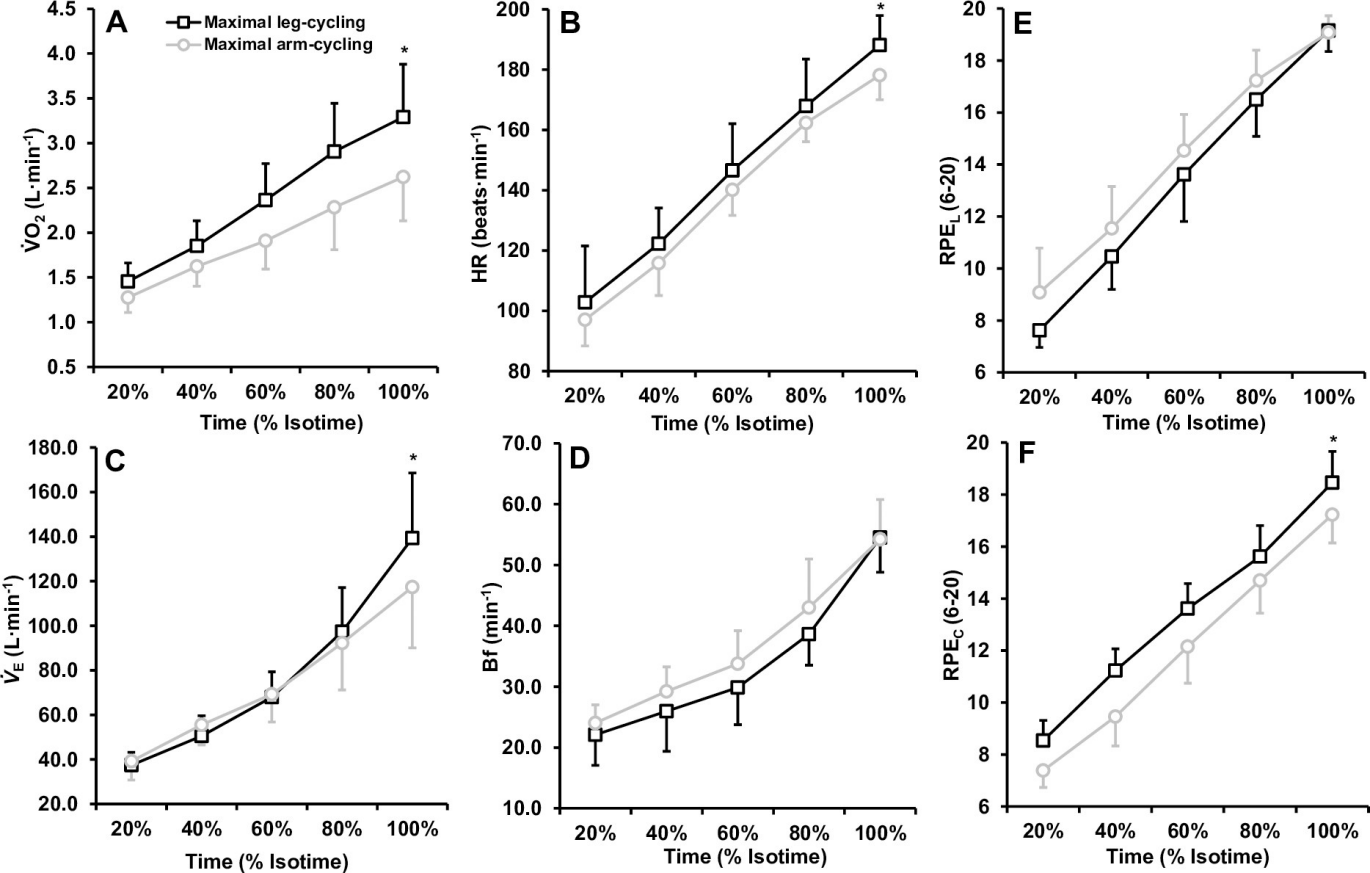
Mean ± SD physiological and perceptual responses to incremental arm-cycling and leg-cycling to volitional exhaustion. Responses are presented at the same time points (20, 40, 60, 80, 100% of the end exercise time). * Indicates significant difference between exercise modes. Note that for all parameters, each isotime point was significantly different to the previous isotime point.

Metabolic, ventilatory, cardiovascular and perceptual responses to maximal arm and leg-cycling are illustrated in Fig 2. The 2 (mode) × 5 (% isotime) way ANOVA’s revealed significant interactions for $\dot{V}O_{2}$ (F_(4,48)_ = 16.363, p < 0.001, *η*^2^ = .577) and ${\dot{V}}_{E}$ (F_(4,48)_ = 20.615, p < 0.001, *η*^2^ = .632). The ANOVA’s also revealed main effects of time for HR, B_f_, RPE_L_ and RPE_C_ (all p < 0.001). Follow up *post-hoc* analysis revealed that each isotime point was significantly greater to the next for both arm and leg-cycling (Fig 2). Additional *post-hoc* analyses revealed that, with the exception of Bf and RPE_C_, upon reaching volitional exhaustion, all variables were statistically greater for leg-cycling compared to arm-cycling (Table 1).

**Table 1:** Maximal cardiorespiratory and perceptual responses to arm-cycling and leg-cycling.

	Arm-cycling (Mean ± SD)	Leg-cycling (Mean ± SD)	p	*d*
$\dot{V}O_{2}{}_{\max}$ (L·min^-1^)	2.62 ± 0.62	3.27 ± 0.61	0.005	1.06
$\dot{V}O_{2}{}_{\max}$ (ml/min/kg)	34.4 ± 5.5	44.3 ± 7.4	0.002	1.53
Maximal Power Output (*W*_max_)	82 ± 18	240 ± 53	0.001	3.99
${\dot{V}}_{E}{}_{\max}$ (L·min^-1^)	117.4 ± 27.3	139.3 ± 29.3	0.001	0.78
B_f_ (breaths·min^-1^)	54 ± 6	55 ± 7	0.877	0.20
HR_max_ (beats·min^-1^)	178 ± 8	188 ± 10	0.003	1.11
RPE_L_	20 ± 1.0	20 ± 1.0	0.819	0.10
RPE_C_	17 ± 1.0	18 ± 1.0	0.001	1.07

**Note** *d*: Cohen’s *d* effect size, p: Alpha value

### Submaximal physiological responses

**Fig 3:**
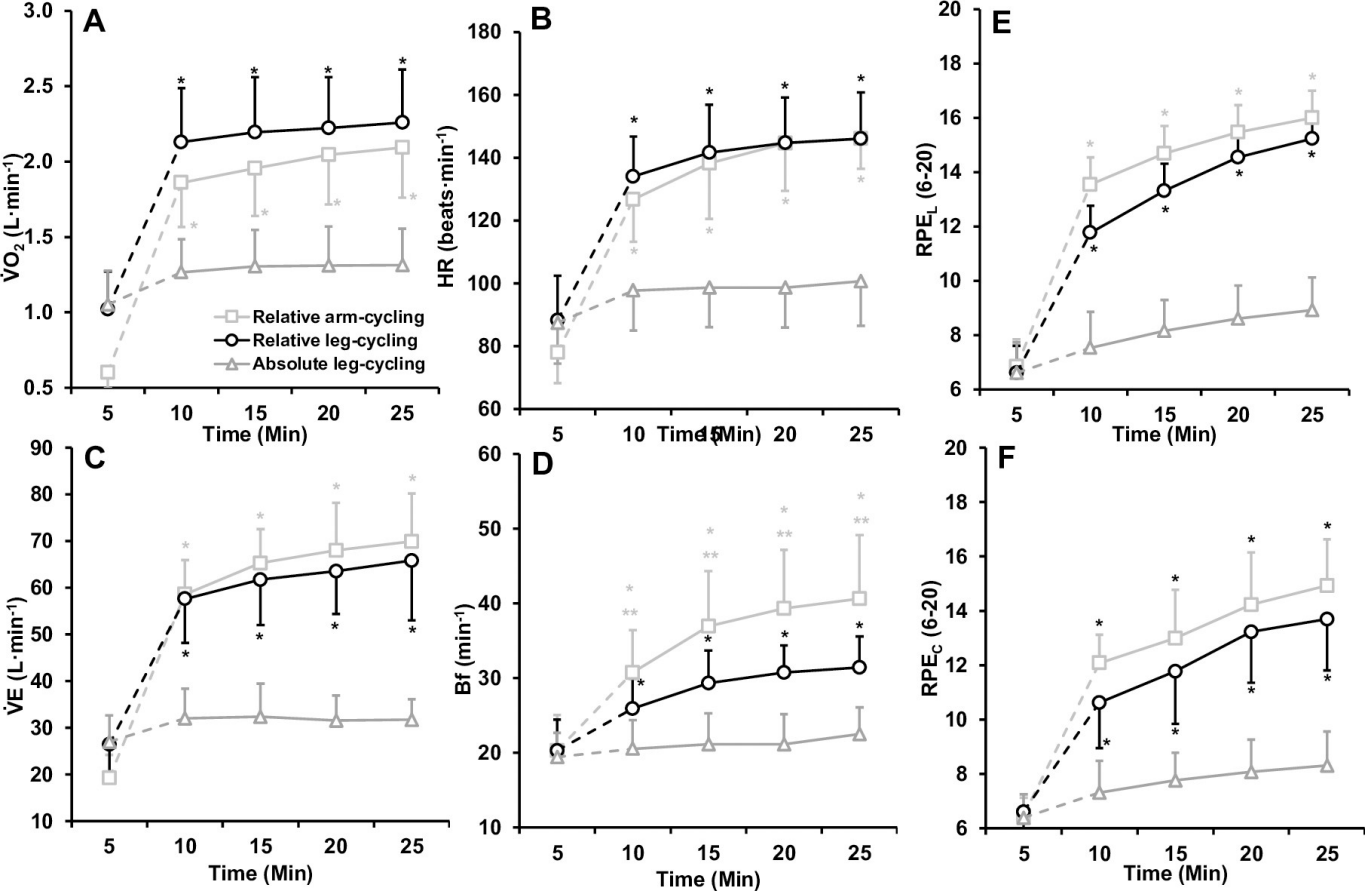
Mean ± SD physiological and perceptual responses to 20-min submaximal arm-cycling and leg cycling to volitional exhaustion. Note that time point 5 to 10 representants the transition from the warm-up to the prescribed workload. * Indicates significant difference to absolute intensity leg-cycling. ** Indicates significant difference to relative intensity leg-cycling.

For the submaximal trials, the 3 (trial) × 5 (time) way ANOVA’s revealed significant interactions for HR (F_(8,96)_ = 63.603, p < 0.001, *η*^2^ = .841), $\dot{V}O_{2}$ (F_(8,96)_ = 35.124, p < 0.001, *η*^2^ = .745), ${\dot{V}}_{E}$ (F_(8,96)_ = 54.141, p < 0.001, *η*^2^ = .819) and Bf (F_(8,96)_ = 21.120, p < 0.001, *η*^2^ = .638) (Fig 3). Follow up *post-hoc* analysis revealed that across each time point $\dot{V}O_{2}$, ${\dot{V}}_{E}$ and HR were statistically greater during relative intensity leg-cycling and relative intensity arm-cycling compared to absolute intensity leg-cycling. There were no statistical differences in any physiological responses between relative intensity arm and leg-cycling (p > 0.05). However, Bf was greater during relative-intensity arm-cycling compared to both leg-cycling trials (p < 0.05). Further, Bf was greater during relative intensity leg-cycling compared to absolute intensity leg-cycling (p < 0.05).

### Executive function following maximal exercise

**Fig 4:**
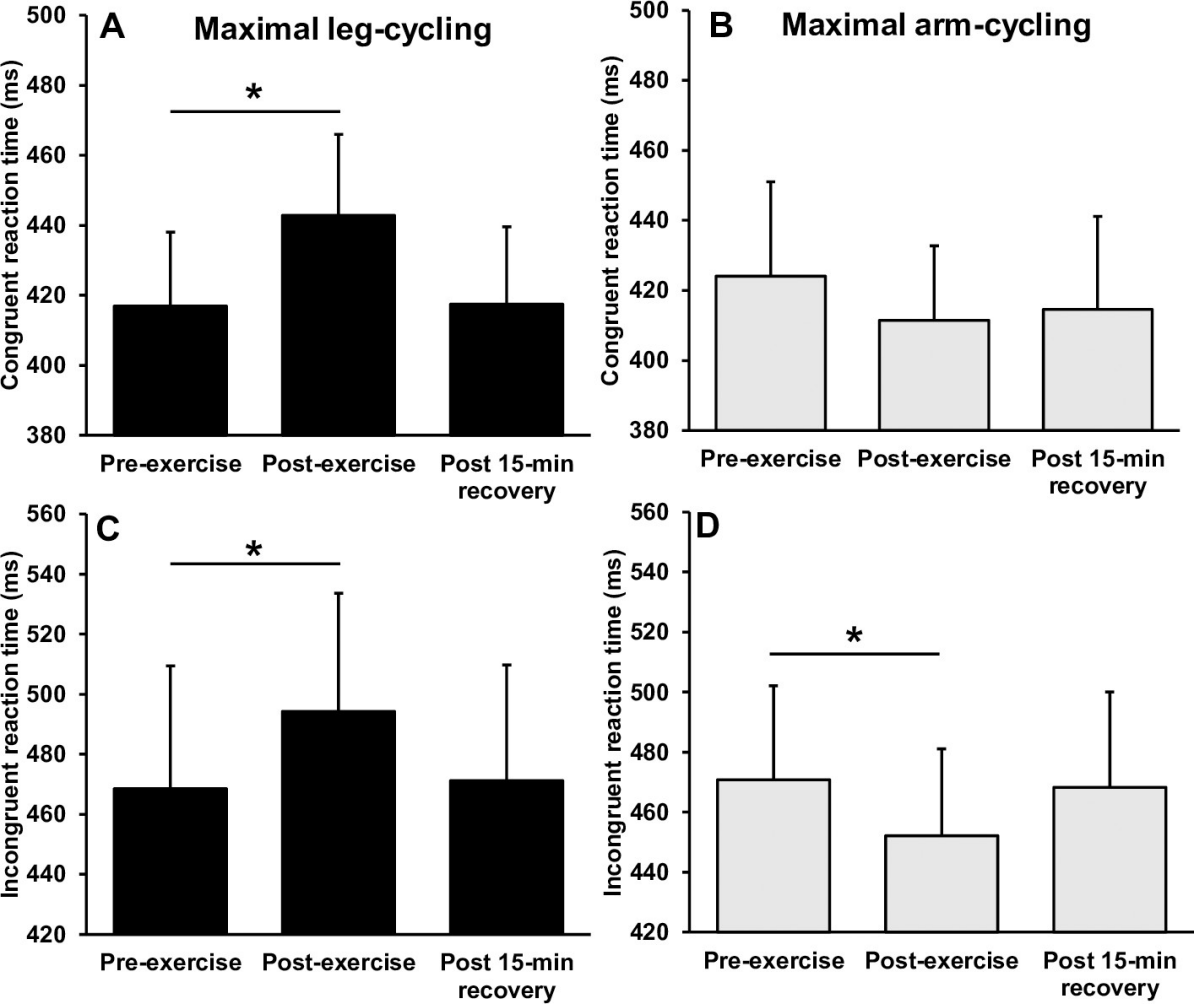
Mean ± SD reaction time before, immediately after and following a 15-min recovery from incremental arm-cycling and leg cycling to volitional exhaustion. * Indicates significant difference to pre-exercise.

**Fig 5:**
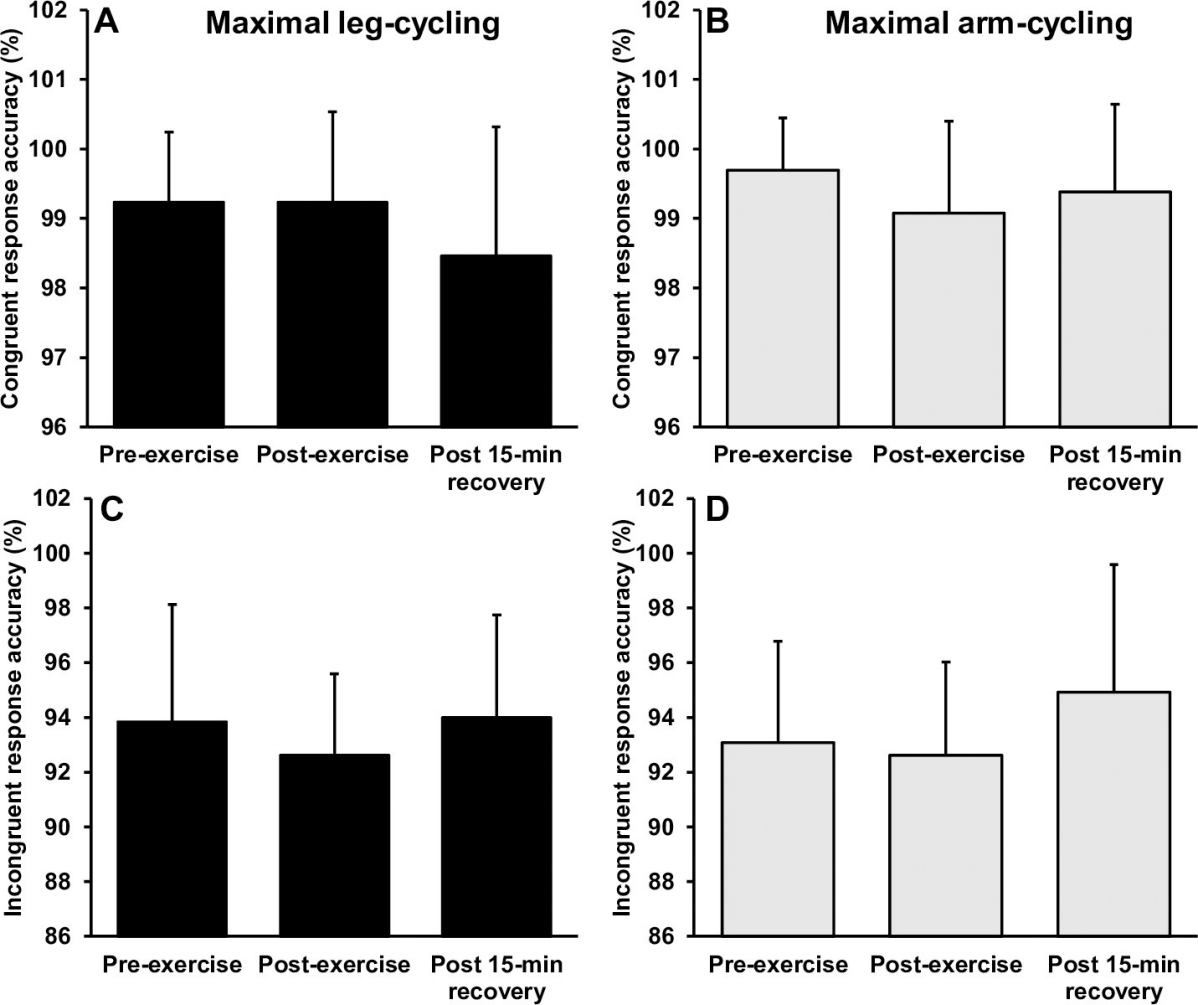
Mean ± SD response accuracy before, immediately after and following a 15-min recovery from incremental arm-cycling and leg cycling to volitional exhaustion. * Indicates significant difference to pre-exercise.

Fig 4 illustrates the reaction times before and after maximal arm and leg-cycling. Separate 2 (mode) × 3 (time) way repeated measures ANOVA’s revealed significant interactions for reaction time during congruent (F_(2,24)_ = 13.848, p = 0.001, *η*^2^ = .536) and incongruent (F_(2,24)_ = 16.386, p = 0.001, *η*^2^ = .577) conditions. For the congruent trials, follow up *post-hoc* analyses revealed a significant and large magnitude increase in reaction time immediately following maximal leg-cycling (p < 0.001, *d* = 1.17), returning to baseline levels after 15-min of recovery (p > 0.05). There was no statically significant change in congruent reaction time following maximal arm-cycling (p = 0.251), although there was a moderate magnitude reduction in reaction time (*d* = -0.52). For incongruent trials, there was a significant and moderate magnitude reduction in reaction time immediately following maximal arm-cycling (p *=* 0.001, *d* = -0.62), returning to baseline levels after 15-min of recovery (p > 0.05). In contrast, there was a moderate and significant increase in reaction time immediately following maximal leg-cycling (p = 0.028, *d* = 0.64), returning to baseline levels after 15-min of recovery (p > 0.05). There were no interactions or main effects observed for response accuracy (p > 0.05) (Fig 5).

### Executive function following submaximal exercise

**Fig 6:**
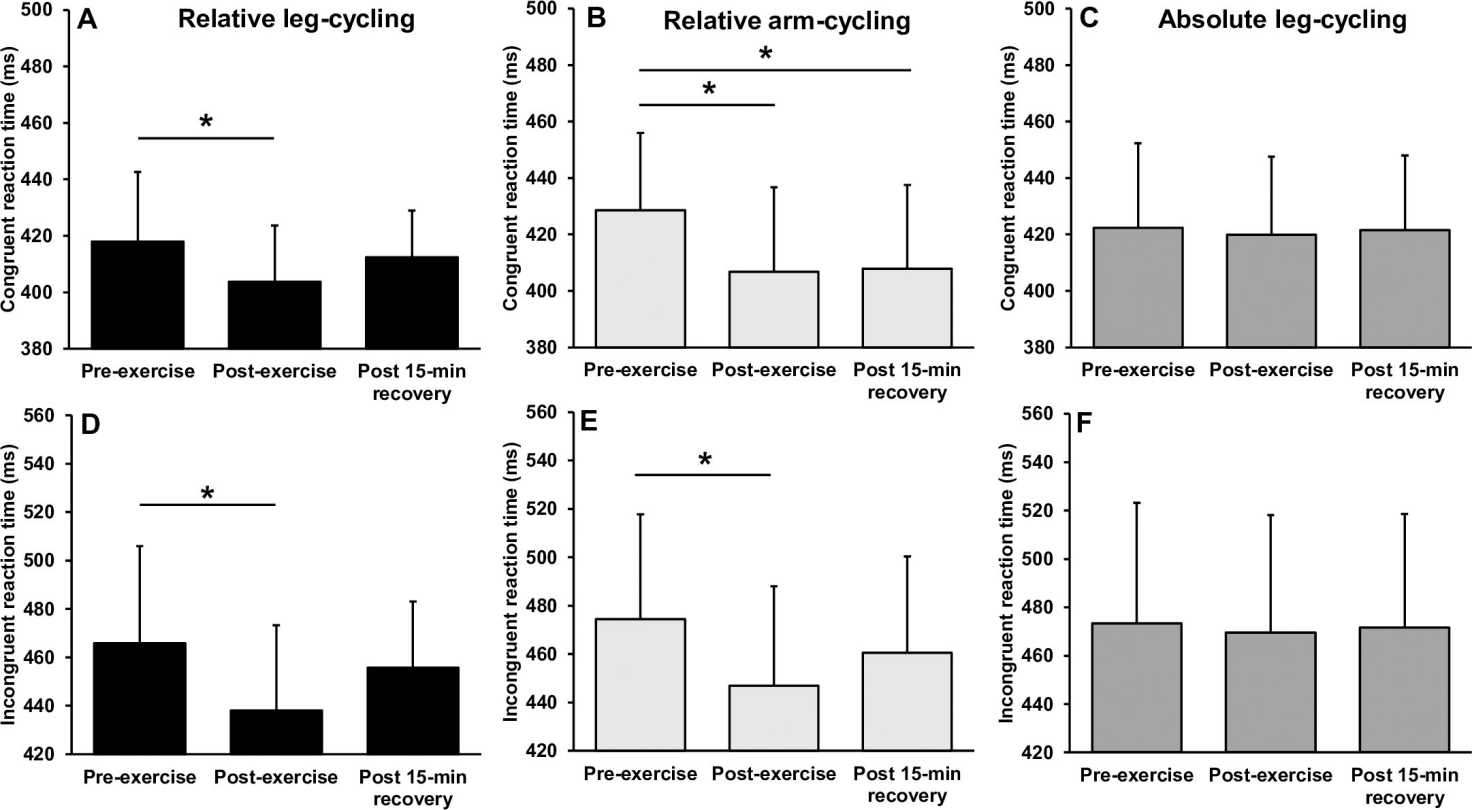
Mean ± SD reaction time before, immediately after and following a 15-min recovery from submaximal arm-cycling and leg-cycling. * Indicates significant difference to pre-exercise.

Fig 6 illustrates the reaction times before and after submaximal arm and leg-cycling. Separate 3 (mode) × 3 (time) way repeated measures ANOVA’s revealed a significant interaction for reaction time during congruent (F_(4,48)_ = 3.300, p = 0.018, *η*^2^ = .216) and incongruent (F_(4,48)_ = 4.311, p = 0.005, *η*^2^ = .264) conditions. For the congruent trials, follow up *post-hoc* analyses revealed a significant and moderate magnitude reduction in reaction time immediately following relative intensity leg-cycling (p = 0.009, *d* = -0.64), returning to baseline levels after 15-min of recovery (p > 0.05). Similarly, a significant and moderate magnitude reduction in reaction time was observed immediately following relative intensity arm-cycling (p = 0.001, *d* = -0.76), remaining significantly faster following 15-min of recovery (p = 0.008, d = -0.73). There was no statistical change in congruent reaction time following absolute intensity leg-cycling (both p > 0.05).

**Fig 7:**
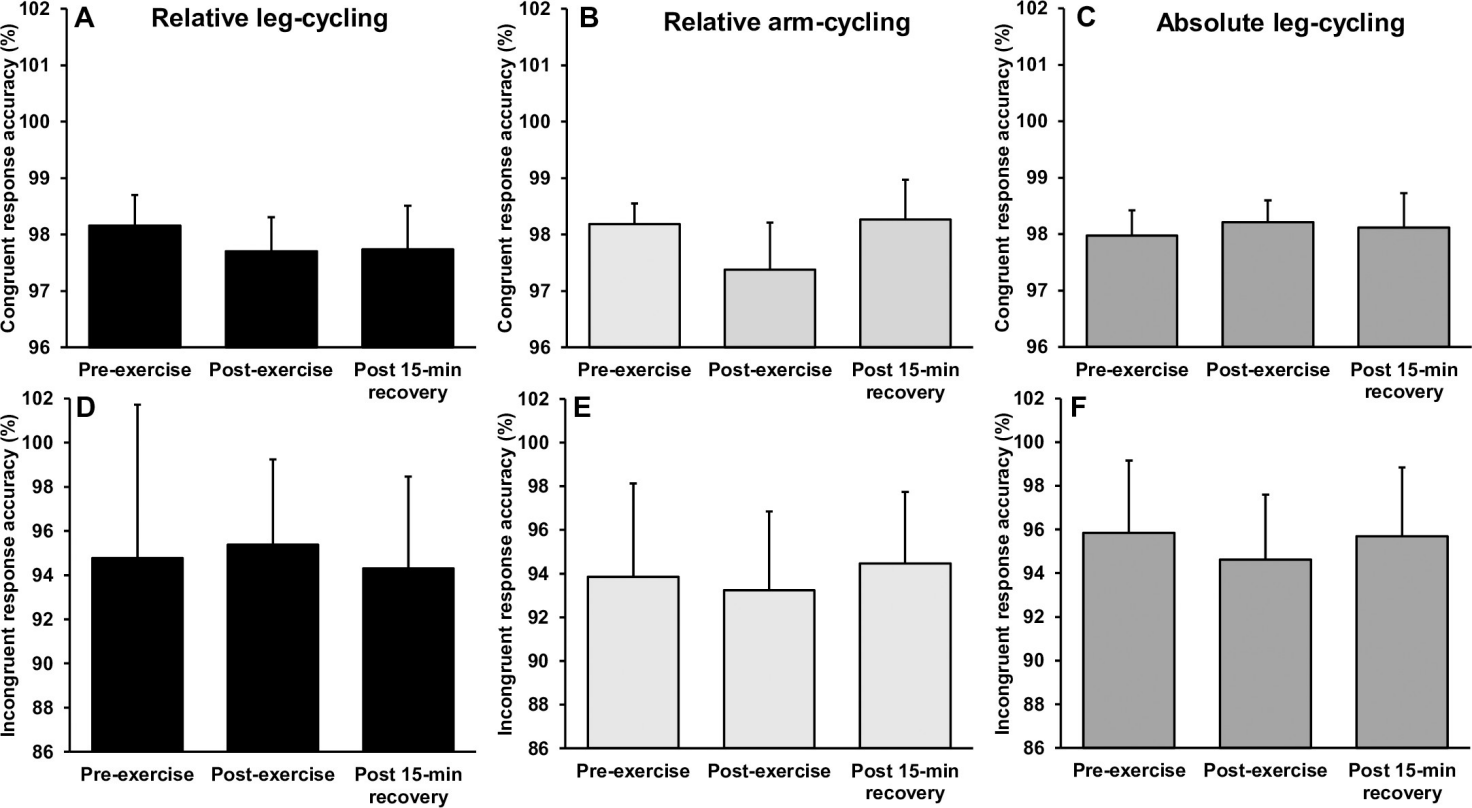
Mean ± SD response accuracy before, immediately after and following a 15-min recovery from submaximal arm-cycling and leg-cycling. * Indicates significant difference to pre-exercise.

For incongruent trials, there was a significant and moderate magnitude reduction in reaction time immediately following relative leg-cycling (p *=* 0.001, *d* = -0.73), returning to baseline levels after 15-min of recovery (p > 0.05). Similarly, a significant and moderate magnitude reduction in reaction time was observed immediately following relative intensity arm-cycling (p = 0.001, *d* = -0.65), returning to baseline levels after 15-min of recovery (p > 0.005). As with the congruent condition, there was no change in incongruent reaction time following absolute intensity leg-cycling (p > 0.05). There were no interactions or main effects observed for response accuracy during congruent or incongruent trials (p > 0.05) (Fig 7).

## Discussion

This is the first study to investigate and compare the effects of acute maximal and moderate intensity arm and leg exercise on executive function. The results of the present study revealed three main findings with respect to understanding the effects of arm compared to leg exercise on cognitive performance; (1) maximal leg-cycling elicited a poorer reaction time, while maximal arm-cycling facilitated reaction time, independent of accuracy (2) arm and leg-cycling performed at the same relative intensity (50% *W*_max_) elicited similar reductions (i.e. faster) in reaction time, without affecting accuracy (3) leg-cycling at the same absolute intensity as arm-cycling did not elicit any statistical changes in cognitive performance. This work shows for the first time that moderate intensity arm and leg-cycling performed at the same relative intensity can elicit similar benefits to executive function, which is of practical importance for those who are restricted to upper limb exercise.

### Executive function performance following maximal incremental exercise

Although many studies have reported changes in cognitive performance immediately following leg-cycling to volitional exhaustion (i.e. [38–42]), results from the present study are unique in that no studies that have compared cognitive performance before and after maximal arm and leg-cycling. Consistent with the literature, the present study found a small, but significant increase (i.e. slower) in reaction time immediately following leg-cycling to volitional exhaustion, returning to pre-exercise levels within 15-min of exercise cessation [38,39]. The worsening of reaction time immediately following exhaustive leg-cycling could be due to a reduction in blood flow to frontal cerebral structures [43], which directly governs the reduction in prefrontal cortex oxygenation [44–46]. The latter is important because impaired cognitive performance during heavy exercise has been linked with a decrease in cerebral oxygenation [47] and recovery of prefrontal oxygenation affects executive function (reaction time) after exhaustive leg-cycling [41]. It is possible that brain neurotransmitters play a key role in speed of processing after exercise. Importantly, the turnover of several neurotransmitters appears to be altered by hypoxia [48], suggesting that oxygen availability is critical for the turnover of neurotransmitters [41]. It can be speculated that oxygen availability was compromised in the brain areas following leg-cycling to exhaustion, which effected the turnover of neurotransmitters and impaired speed of processing.

Following maximal arm-cycling we found significant improvements (i.e. faster) in reaction time. It is plausible that sufficient oxygen availability after exhaustive arm exercise may have maintained speed of processing. For example, the $\dot{V}O_{2}{}_{\max}$ achieved during arm-cycling was 81% of that achieved during leg-cycling. The lower maximal responses (HR_max_, $\dot{V}O_{2}{}_{\max}$, ${\dot{V}}_{E}{}_{\max}$) observed during arm-cycling are a result of peripheral factors limiting exercise such as the utilisation of a relatively small muscle mass when rather than cardiorespiratory parameters (i.e. with leg-cycling) [18] and point towards lower levels of exercise-induced arousal during maximal arm compared to leg-cycling. Another explanation for the differential effects of exhaustive arm and leg-cycling on cognitive performance might be that the attentional demands might be greater during leg cycling, leaving less attentional resources available for the cognitive task. For example, the cerebral uptake of oxygen and glucose (i.e. the cerebral metabolic ratio [6:1]) decreases in response to exhaustive exercise [49]. The reduction in the cerebral metabolic ratio immediately following exercise to exhaustion points towards an influence of the mental effort associated with the exercise [50]. Although both maximal arm and leg-cycling reduce the cerebral metabolic ratio, the reduction is larger for leg-cycling [23], suggesting that less mental effort is required during maximal arm-cycling. Therefore, the maintenance of cognitive performance following arm-cycling to exhaustion may be related to lower mental effort associated with arm exercise. Furthermore, during dynamic exercise engaging a smaller active muscle mass, less extraneous sensory information is processed [51]. Therefore, attentional demands might be lower during arm-cycling, allowing greater attentional resources for the cognitive task [3].

### Executive function performance following submaximal exercise

Consistent with previous literature, moderate intensity leg-cycling facilitated executive function through a reduction (i.e. faster) in reaction time, with no difference in response accuracy [2–5]. The arm-cycling modality investigated in the present study further allowed us to determine whether this type of exercise adds value as an exercise intervention given that leg-cycling has been extensively studied. To the authors knowledge, this study is the first to investigate cognitive performance after moderate intensity arm and leg exercise. The present results show that arm-cycling matched at the same relative intensity as leg-cycling (i.e. 50% *W*_max_) elicited comparable improvements in reaction time, without losses in accuracy. From a practical perspective, these findings have important implications as they suggest that individuals restricted to upper body exercise can achieve a similar exercise-induced improvement in cognitive performance as those able to perform lower body exercise. The similar improvements in post-exercise cognitive performance following relative intensity arm and leg-cycling are likely explained by similar metabolic ($\dot{V}O_{2}$), cardiovascular (HR) and ventilatory (${\dot{V}}_{E}$) stimulus during these protocols and would seemingly yield similar increases in arousal [3]. In contrast, there was no change in cognitive performance following leg-cycling at the same absolute intensity (external workload) as relative intensity arm-cycling, presumably due to limited activation in the relevant brain areas [5]. This result is commensurate with other studies using low exercise intensities (i.e. 40% *W*_max_) [52]. Although exercising at different intensities for the same amount of time elicits different energy demands, which might theoretically influence cognitive processing [3], we acknowledge that our markers of exercise intensity (i.e. cardiorespiratory measures), and thus arousal, are unlikely to precisely represent changes occurring in the relevant brain areas.

Several biological mechanisms have been offered to explain improvements in cognitive performance following moderate intensity aerobic exercise. Firstly, exercise intensities ranging from 48–60% $\dot{V}O_{2}{}_{\max}$ (similar to the relative intensity arm and leg trials in the present study) elicit an increase in cerebral blood flow [12,53] which is related to increased neuronal activity in the prefrontal cerebral cortex [49], and subsequent improvement in executive function [25]. In the present study, the null findings with respect to changes in cognitive performance following absolute intensity leg-cycling might be related to the lower cardiac output during this exercise (~39% $\dot{V}O_{2}{}_{\max}$). For example, cardiac output has been shown to have a linear relationship with cerebral blood flow [53]. Although cardiac output was not measured in the present study, HR was significantly higher during relative intensity arm-cycling compared to leg-cycling at the same absolute intensity. From a psychophysiological perspective, moderate intensity exercise elicits an increase in brain concentrations of norepinephrine, dopamine, adrenocorticotropin hormone and cortisol (i.e. optimal performance) [4]. There is evidence that arm-cycling may elicit a greater catecholamine output compared to leg-cycling at a given oxygen uptake [29]. More recently, Leicht et al. [21] reported that arm and leg-cycling for 45-min at 60% $\dot{V}O_{2}{}_{\max}$ (relative intensity comparison) elicited the same epinephrine response, while cycling at the same absolute intensity as arm-cycling resulted in a blunted increase in epinephrine. This is important because increased catecholamine concentrations signify increased arousal, which should theoretically improve speed of processing by vagal/nucleus tractus solitarii pathway activation and central increases due to perceptions of stress [11]. From a cognitive psychology perspective, exercise is viewed as a stressor which, as intensity increases, leads to increased levels of arousal [5]. Common to “arousal” theories [15,54] is the assumption that cognitive performance is dependent on the allocation of energetic resources to meet the task demands. That is, an inverted-U effect of exercise on cognitive performance would be demonstrated with low intensity exercise (low arousal) inducing poor cognitive performance (i.e. absolute intensity leg cycling), moderate intensity (optimal arousal) eliciting peak cognitive performance (i.e. relative intensity arm and leg-cycling) and exhaustive exercise (high arousal) inducing poor cognitive performance (i.e. maximal leg-cycling) [3].

A previous meta-analysis reported suggested that the greatest beneficial effects of moderate-intensity exercise occurs 11 to 20 minutes after exercise cessation [2]. In the present study, the beneficial effects of leg-cycling had dissipated within 15-min of exercise cessation. In contrast, following arm-cycling, cognitive performance remained significantly improved following 15-min recovery (congruent trials only). These findings might have important practical implications. For example, if arm-cycling is adopted to improve cognitive performance of clinical populations with limited lower body exercise capacity, such as those with neurological disorders, or patients with lower limb peripheral arterial disease, we show that there is a window of opportunity following acute upper body exercise for presenting such groups with tasks that challenge executive function.

### Strengths and limitations

This is the first investigation to attempt to identify differences in the effects of upper versus lower body exercise on cognitive performance. This study is of practical importance because leg training fails to accommodate individuals who are unable to perform sustained lower limb exercise, such as those lower-limb orthopaedic problems, neurological disorders or peripheral arterial disease. Additionally, the majority of the evidence for a beneficial effect of acute exercise on cognitive performance utilized leg-cycling or treadmill protocols. Another novelty and strength of this study was that submaximal leg-cycling trials were performed at the same relative (% *W*_max_) and absolute (W) intensity as arm-cycling. This study design allowed us to determine whether changes in cognitive performance were specific to the active muscle mass rather than the physiological exertion experienced. The study was further strengthened by a familiarization session that aimed to eliminate potential learning effects and the within-subject, cross-over design.

The present study has some limitations that should be acknowledged. Firstly, although we offered several mechanisms that affect cognitive performance, we were unable to take measures offering further insight into cerebral changes, which might elucidate the mechanisms underlying the differential effects of arm and leg-cycling on cognitive performance. Secondly, in the present study, we focused on how alternations in cardiorespiratory variables affect executive function. Accordingly, we acknowledge that our markers of exercise intensity (i.e. cardiorespiratory measures), and thus arousal, are unlikely to precisely represent changes occurring in the relevant brain areas following exercise. Thirdly, although this study extends the previous findings by comparing arm and leg-cycling on cognitive performance, owing to methodologic limitations (i.e. use of the hands during arm-cycling), only post-exercise cognitive performance was examined. The problems with testing post-exercise cognitive performance have long been documented [55], as some individuals, particularly those with high levels of physical fitness, recover quickly [56]. The results of the present study should therefore not be generalized to cognitive performance during exercise. To accurately couple the exercise load/stimulus with the cognitive response it is important to ensure that the cognitive testing occurs at the same time as the exercise. This was not possible in the case of arm-cycling and the Flanker task, due to the requirement to use both hands for both tasks. Future work might therefore look to examine other types of cognition which do not require use of the hands, such as auditory or visually related cognitive tasks. Finally, our sample size was limited and included only males, which precludes us from generalizing our findings to females or different age groups. However, the authors anticipate that this exploratory study will provide the impetus for further trials involving a larger sample size to more accurately quantify exercise-induced changes in cognitive performance following arm and leg-cycling.

## Conclusion

We initially hypothesized that improvements in executive function would be greater following arm-cycling than leg-cycling at the same relative intensity. Instead, this study showed that acute arm-cycling performed at the same relative intensity than leg-cycling elicited comparable improvements in cognitive function. Therefore, these findings do not support the first hypothesis. With respect to our second hypothesis, we confirmed that arm-cycling elicited greater improvements in executive function than leg-cycling at the same absolute intensity. Finally, we found that maximal leg-cycling elicited a poorer reaction time, while maximal arm-cycling facilitated reaction time. This finding does not support the final hypothesis. These findings suggest that individuals restricted to arm exercise possess a similar capacity to elicit an exercise-induced cognitive performance benefit, which might lead to the development and optimization of exercise interventions to improve cognitive function.

## Supporting information

S1 Table (PDF)
