# Supplementary material for: Exercise intensity-dependent effects of arm and leg-cycling on cognitive performance
Source: PLoS One. 2019 Oct 21;14(10):e0224092. doi: 10.1371/journal.pone.0224092 (PMC6802839; doi:10.1371/journal.pone.0224092)
Supplement: S1 Table — (PDF) [file pone.0224092.s001.pdf]

Reaction Time (ms)					
Maximal leg cycling			Maximal arm cycling		
Pre_CON	Post_CON	Recov_CON	Pre_CON	Post_CON	Recov_CON
440.88	453.4	421.5	464.5	426.68	438.26
435.08	453	441.54	424.32	426.1	411.46
408.54	420	408.58	391.88	384.26	372.98
423.1	454.48	427.84	410.56	416.3	412.02
419.78	426.18	416.08	417.6	433.64	443.76
444.98	468	463.7	434.9	429.54	437.76
386.38	408.94	410.1	405.64	422	414.7
384.6	410.42	400.06	415.28	400.22	371.32
424.16	463.86	431.42	455.62	427.24	443.48
399.32	434	376.56	394.8	387.2	387
434.76	469	420.52	416.32	374.8	408.26
389.22	423.28	390.66	402.92	388.56	400.46
428.56	472.36	418.26	478.58	432.98	447.54

Reaction Time (ms)					
Maximal leg cycling			Maximal arm cycling		
Pre_INCON	Post_ INCON	Recov_ INCON	Pre_ INCON	Post_ INCON	Recov_ INCON
440.88	453.4	421.5	464.5	426.68	438.26
435.08	453	441.54	424.32	426.1	411.46
408.54	420	408.58	391.88	384.26	372.98
423.1	454.48	427.84	410.56	416.3	412.02
419.78	426.18	416.08	417.6	433.64	443.76
444.98	468	463.7	434.9	429.54	437.76
386.38	408.94	410.1	405.64	422	414.7
384.6	410.42	400.06	415.28	400.22	371.32
424.16	463.86	431.42	455.62	427.24	443.48
399.32	434	376.56	394.8	387.2	387
434.76	469	420.52	416.32	374.8	408.26
389.22	423.28	390.66	402.92	388.56	400.46
428.56	472.36	418.26	478.58	432.98	447.54

Response accuracy (%)					
Maximal leg cycling			Maximal arm cycling		
Pre_CON	Post_CON	Recov_CON	Pre_CON	Post_CON	Recov_CON
100	100	100	100	100	100
98	100	96	100	96	100
100	100	100	100	100	100
100	100	100	100	100	100
100	96	96	100	100	100
98	100	100	100	100	100
98	98	100	100	98	100
98	100	96	98	98	96
100	100	98	98	100	100
100	100	96	100	98	98
100	98	98	100	100	100
100	100	100	100	100	100
98	98	100	100	98	98

Response accuracy (%)					
Maximal leg cycling			Maximal arm cycling		
Pre_INCON	Post_ INCON	Recov_ INCON	Pre_ INCON	Post_ INCON	Recov_ INCON
98	94	98	94	94	100
88	92	90	90	96	94
94	98	94	98	92	92
98	92	96	92	96	98
88	88	88	90	88	100
96	90	94	88	90	92
90	90	94	96	96	98
88	90	92	88	86	88
98	96	100	96	92	98
100	94	94	100	96	96
94	90	88	92	90	86
96	94	98	94	96	92
92	96	96	92	92	100

Reaction Time (ms)								
Relative leg cycling			Relative arm cycling			Absolute leg cycling		
Pre_CON	Post_CON	Recov_CON	Pre_CON	Post_CON	Recov_CON	Pre_CON	Post_CON	Recov_CON
426.4	412.56	399.4	422.24	407	422.68	436.44	423.12	427.32
436.38	420	413.86	435.7	416.12	414	437.38	442.2	444.94
390.52	396.32	382.98	399.28	386	403	381.54	388.08	396.24
402.78	404.98	413.24	423.58	402.32	416.2	439.98	429.54	422.54
425.38	406.76	406.94	402.24	390	397.6	439.5	429	420.34
424.04	403	434.52	479.56	447	455.5	426.42	464.94	451.84
410.92	396	428.94	443.4	423.84	404.7	415.34	417.24	444.54
421.36	377.02	421.99	402.86	347	340	395.14	394.02	375.62
463.66	444.66	438.96	472.28	442.28	424	462.88	457.72	444.3
377.62	361.54	392.08	397.26	379.28	373.04	373.56	373.25	371.14
429.32	414.38	414.38	427	390.72	404.54	458.48	404.9	424.38
382.94	403.4	397.84	412.54	404.48	398.96	383.1	395.48	413.08
443	408	416	454.26	452.8	448.48	441.5	439	443.64

Reaction Time (ms)								
Relative leg cycling			Relative arm cycling			Absolute leg cycling		
Pre_INCON	Post_ INCON	Recov_ INCON	Pre_ INCON	Post_ INCON	Recov_ INCON	Pre_ INCON	Post_ INCON	Recov_ INCON
452.22	421	447.08	467.8	454	485.58	451.18	468.08	479.14
483.5	476	463.8	484.64	442.18	460.64	471.4	488.36	492.84
428.04	401	422.18	447.52	402.02	454.5	439.62	435	421.42
455.64	425	468.28	473.02	422	459.9	503.52	483.84	491.48
459.3	445.8	457.26	443.38	447.2	432.78	485.7	484	473.76
502.96	466.2	490.76	529.54	501.44	517.82	499.68	514.36	501.38
443.88	436	467.5	520.22	457.04	446.82	479.84	491.64	484.32
459.44	414.24	422.28	409	396	425.02	421.22	412.92	413.68
505.92	471.9	486.96	501.46	483.8	498.8	536.16	497.6	510.88
394.16	361.32	404.38	409.54	388.12	383.14	408.1	385.22	406
533.54	467.54	467.54	483.32	463	443.6	478.16	452.2	451.24
423.06	424	437	449.24	426	445.92	403.8	420.54	430.82
513.5	484	488	549.38	527	531.56	575.78	570	575

Response accuracy (%)								
Relative leg cycling			Relative arm cycling			Absolute leg cycling		
Pre_CON	Post_CON	Recov_CON	Pre_CON	Post_CON	Recov_CON	Pre_CON	Post_CON	Recov_CON
99	98	98	98	98	99	98	99	99
97	98	98	98	98	99	98	99	99
99	98	98	98	98	99	98	99	99
98	98	98	98	98	99	98	98	99
98	98	97	98	98	99	98	98	98
98	98	99	98	97	98	98	98	98
98	98	99	98	97	98	97	98	98
98	98	98	98	97	98	97	98	98
98	98	97	98	97	98	99	99	98
98	98	98	98	97	97	98	98	98
99	97	99	99	97	97	98	98	98
99	97	97	99	96	99	98	98	97
98	96	96	98	96	99	98	97	97

Response accuracy (%)								
Relative leg cycling			Relative arm cycling			Absolute leg cycling		
Pre_INCON	Post_ INCON	Recov_ INCON	Pre_ INCON	Post_ INCON	Recov_ INCON	Pre_ INCON	Post_ INCON	Recov_ INCON
94	98	96	98	96	98	100	94	96
94	90	86	88	88	90	98	92	92
98	96	94	94	98	94	90	92	96
98	100	98	98	96	96	98	100	100
96	88	92	88	86	88	98	96	96
100	96	96	96	94	94	98	96	92
92	94	88	90	90	94	94	100	94
76	92	92	88	90	92	90	90	92
98	98	98	98	96	100	98	94	98
100	98	98	100	94	94	96	94	92
86	92	92	94	94	94	92	92	96
100	98	96	96	94	98	96	94	100
100	100	100	92	96	96	98	96	100
